# Effects of Dicationic Imidazolium-Based Ionic Liquid Coatings on Oral Osseointegration of Titanium Implants: A Biocompatibility Study in Multiple Rat Demographics

**DOI:** 10.3390/genes13040642

**Published:** 2022-04-02

**Authors:** Sutton E. Wheelis, Claudia C. Biguetti, Shruti Natarajan, Bhuvana Lakkasetter Chandrashekar, Alexandra Arteaga, Jihad El Allami, Gustavo P. Garlet, Danieli C. Rodrigues

**Affiliations:** 1 Department of Bioengineering, The University of Texas at Dallas, Richardson, TX 75080, USA; sxw110230@utdallas.edu (S.E.W.); claudia.biguetti@utdallas.edu (C.C.B.); bhuvana.lakkasetterchandrashekar@utdallas.edu (B.L.C.); alexandra.arteaga@utdallas.edu (A.A.); jihad.allami@utdallas.edu (J.E.A.); 2 Department of Biological Sciences, The University of Texas at Dallas, Richardson, TX 75080, USA; shruti.natarajan@utdallas.edu; 3 Texas A&M College of Dentistry, Dallas, TX 75246, USA; 4 Department of Biological Sciences, Bauru School of Dentistry, University of São Paulo, São Paulo 01000, Brazil; garletgp@usp.br

**Keywords:** ionic liquids, titanium, osseointegration, multifunctional coatings, biocompatibility

## Abstract

Dicationic imidazolium-based ionic liquids with amino acid anions, such as IonL-phenylalanine (IonL-Phe), have been proposed as a multifunctional coating for titanium (Ti) dental implants. However, there has been no evaluation of the biocompatibility of these Ti coatings in the oral environment. This study aims to evaluate the effects of IonL-Phe on early healing and osseointegration of Ti in multiple rat demographics. IonL-Phe-coated and uncoated Ti screws were implanted into four demographic groups of rats to represent biological variations that could affect healing: young males (YMs) and females (YFs), ovariectomized (OVXFs) females, and old males (OMs). Samples underwent histopathological and histomorphometric analysis to evaluate healing at 7 and 30 days around IonL-coated and uncoated Ti. The real-time quantitative polymerase chain reaction was also conducted at the 2- and 7-day YM groups to evaluate molecular dynamics of healing while the IonL-Phe was present on the surface. IonL-coated and uncoated implants demonstrated similar histological signs of healing, while coated samples’ differential gene expression of immunological and bone markers was compared with uncoated implants at 2 and 7 days in YMs. While YMs presented suitable osseointegration for both uncoated and IonL-Phe-coated groups, decreased success rate in other demographics resulted from lack of supporting bone in YFs and poor bone quality in OVXFs and OMs. Overall, it was found that IonL-coated samples had increased bone-to-implant contact across all demographic groups. IonL-Phe coating led to successful osseointegration across all animal demographics and presented the potential to prevent failures in scenarios known to be challenged by bacteria.

## 1. Introduction

Titanium remains the most popular and successful biomaterial utilized in dental implant fixtures because of its mechanical properties and biocompatibility [1]. This success is dependent on the formation and maintenance of osseointegration—the growth and mechanical anchorage of patient alveolar bone into the threads of the implant [2,3]. For over 50 years, dental implants have been widely utilized as a functional substitute for teeth, as it is estimated that 5.7% of adults with edentulism have received at least one implant from 2015 to 2016 in the United States, with that rate expected to increase up to 23% by 2026 [4,5]. Although the device reported has success rates from 90 to 98%, recent systematic reviews indicate that an average of 2.7% of implants fail within one year, and 3.6% fail after ten years in healthy adults [6,7,8,9,10,11,12]. These rates can increase when considering the possible impacts of patient biological variations such as older age [8,9] and gender [10,11]. Therefore, there is still a need for therapeutic interventions aimed at increasing the predictability and long-term maintenance of osseointegration in both healthy patients and those with systemic conditions that can affect their chances of implant success. First, the etiology of failures must be understood. Dental implant failures are characterized by the absence or disruption of osseointegration, and their causes are often multifactorial, involving surgical trauma, instability, infection, and poor bone quality and quantity [12,13,14,15,16]. Regardless of the cause, the underlying mechanism that leads to implant failure is an unbalanced, destructive inflammatory response that results in fibrosis or chronic matrix breakdown of hard and soft tissue around the implant [17,18].

In order to understand the conditions in which constructive or destructive inflammation is created, the elements of biomaterial–tissue interactions must be discussed. Anytime a biomaterial is placed in a living organism, there are two main contributors that dictate the nature and intensity of the inflammatory response. First, there is the acute and aseptic inflammation initiated by the host from the injury associated with the surgical placement of the implant [19]. Second, there is an additional immunomodulatory influence from the biomaterial as it interacts with host tissue [18,19]. In ideal conditions, the titanium implant is immediately surrounded by blood after placement into the implant bed, resulting in protein adsorption onto the implant surface [19]. The constituents and characteristics of this protein adhesion layer are based on titanium oxide surface chemistry and result in a milieu of blood plasma-derived molecules, cytokines, chemokines, and damage-associated molecular patterns (DAMPs) [19,20,21]. Some DAMPs, such as high mobility group box protein 1 (HMGB1), not only play a role in initiating the innate immune response but perform important immunomodulatory functions related to osseointegration, such as mesenchymal stem cell (MSC) recruitment, primarily through their interactions with macrophages [21,22]. Macrophages are key cells in recognizing components from the protein layer on the titanium surface and determining the outcome of the acute inflammatory response, with the ability to polarize into classical M1 (proinflammatory) or M2 (anti-inflammatory) phenotypes to recruit and direct the cells required for tissue regeneration [23,24,25]. Normal Ti–tissue interactions are characterized by a controlled balance of both M1 and M2 macrophages, polymorphonuclear cells (PMNs), and lymphocytes during the initial immune response, which give way to M2-dominated sequential healing stages that commence MSC recruitment, differentiation, bone formation, and remodeling [23,26]. These desirable interactions can be disrupted by variations in biomaterial surface [27] (corrosion, topography), or by external factors such as bacterial infiltration [28,29], micromotion, luting cement, and various patient-related factors such as low bone quality and quantity [30,31]. As the population of those receiving dental implants are typically at an increased risk for these clinical conditions, efforts have been made to investigate ways to encourage an environment conducive to normal Ti–tissue interactions [32]. 

Bioengineered interventions for dental implants focus on modifying or coating the material’s surface without modifying its bulk properties [1,33]. As discussed, the inflammatory response to an implant is determined by the interplay of several factors. There are a large variety of proposed surface modifications in the literature. Most recently, there have been attempts to develop coatings that address one or more external factors that can interfere with the healing process or modulate the inflammatory response toward more regenerative outcomes [34,35,36,37]. The coatings that address external factors such as bacteria are preventing one mode of destructive inflammation but do not address the impact of underlying patient factors or excessive surgical trauma on healing. Immunomodulatory coatings do take these aspects into account and continue to be the focus of recent literature. This class of coatings can employ simple surface topography/wettability changes or apply a variety of molecules to address specific detrimental patient factors [38,39,40,41,42]. Surface topography/wettability modifications typically aim to encourage M1 to M2 macrophage polarization during the acute healing period to commence MSC recruitment and bone remodeling, but conversely, they can be more prone to bacterial colonization [39]. Additionally, they do not address all patient factors, such as age-related immunosenescence, that can impact the innate proinflammatory processes necessary to initiate healing [43,44]. The application of more biologically active molecules such as alendronic acid, prostacyclin, or insulin-like growth factor 1 (IGF1) can address impaired healing associated with osteoporosis and diabetes in aged patients [45,46,47,48,49,50]. However, care must be taken to make sure that they are not interfering with implant biocompatibility.

As a result, there is currently a need for the development of a biocompatible, multifunctional, and customizable surface modification for dental implants, given the context of the healing environment. Ionic liquids (IonLs) have been previously investigated for their potential as device coatings [51]. IonLs are a customizable class of molten salts that have been utilized to serve a wide variety of functions, such as lubricants [52,53], solvents [54], and active pharmaceutical ingredients [55,56]. Recently, dicationic imidazolium-based IonLs with amino acid anions have emerged as an excellent candidate to address the need for titanium dental implant coatings that are multifunctional and regenerative. Two promising formulations with phenylalanine (IonL-Phe) and methionine (IonL-Met) have been extensively evaluated in vitro [51,57,58,59]. These coatings have demonstrated stable, lubricative, anticorrosive, and antimicrobial activity against several early colonizing strains of oral bacteria on the surface of titanium [57,58,59]. In addition, it was found that these IonLs do not interfere with normal Ti–tissue interactions in a subcutaneous animal model and, in fact, demonstrate release behavior in vitro and in vivo, where IonL cannot be observed 14 days after Ti implantation in surrounding subcutaneous tissue of rats [60]. This could limit potential interference with the normal progression of inflammation and healing while still implementing demonstrated multifunctionality. These findings provide motivation for further evaluation of the coating’s biocompatibility in more complex healing scenarios, such as oral osseointegration. 

As previously mentioned, there is a need for a device coating that has the multifunctionality to address several modes of implant failure while maintaining a regenerative healing response. IonLs have demonstrated multifunctionality in vitro by mitigating conditions that contribute to failure or affect implant performance. Although IonL biocompatibility has been confirmed subcutaneously, Ti–tissue interactions associated with successful osseointegration in the oral environment are more delicate because of exposure to external elements such as bacteria, food bolus occlusal forces, and patient factors previously discussed [60]. Therefore, it is essential to investigate whether IonLs maintain or modulate the inflammatory response into a more constructive or destructive inflammatory scenario and determine whether mucosal and bone integration is affected as a result. The purpose of this study is to assess the effect that IonL coatings have on inflammation, healing, and early intramembranous osseointegration of titanium in vivo to confirm the biocompatible and temporary aspects of the coating in a more clinically relevant model. Establishing the biocompatibility of these coatings is the next step in the effort to develop a failure mitigating, regenerative, and potentially customizable surface for dental implants. This evaluation will utilize a preclinical oral osseointegration model in the Lewis rat. Although rodent models for osseointegration cannot accurately simulate human occlusal loads, they remain a robust preclinical model for early osseointegration, as these animals undergo inflammatory and intramembranous bone remodeling benchmarks similar to humans’ [26,61,62,63]. The particular Lewis rat model utilized in this study has been well characterized for these healing benchmarks during Ti–tissue healing and will allow for a detailed comparison of cellular and molecular mechanisms between IonL-coated and uncoated Ti [62]. In addition, the IonLs will be evaluated in several other demographic groups of Lewis rats to confirm the coating does not have a detrimental effect on osseointegration with animals that possess biological variations that affect bone quality and healing capacity, to simulate clinically relevant patient factors in humans. It is hypothesized that IonL coatings will maintain the biocompatibility of titanium necessary for successful osseointegration in ideal conditions, with the potential to improve the success rate and bone-to-implant contact (BIC%) in other rat demographics with nonideal healing conditions. Following implantation, the effect of IonLs on early healing was assessed using clinical, histological, and molecular characterization to evaluate the impact of coating on healing. 

## 2. Materials and Methods

### 2.1. Selection of Ionic Liquid Formulation

Two formulations of IonL were chosen as candidates for this study because of their previously demonstrated in vitro properties [51,57,58,59] and in vivo biocompatibility in a subcutaneous model [60]: 1,10-bis(3-methylimidazolium-1-yl) decane diphenylalanine (IonL-Phe) and 1,10-bis(3-methylimidazolium-1-yl) decane dimethionine (IonL-Met). IonLs were synthesized based on a method proposed by Fukumoto et al. using protocols previously established in the literature [64]. The IonL were characterized using 1H and 13C nuclear magnetic resonance spectrometry to verify structure with existing literature [51].

**Figure 1:**
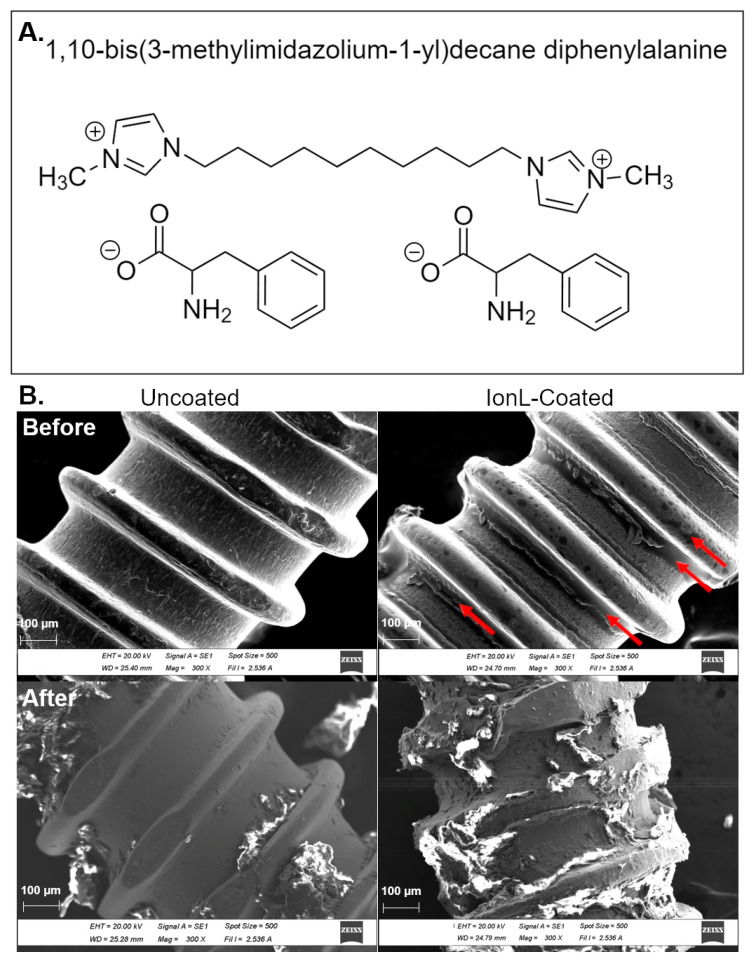
(**A**) Chemical structure of IonL-Phe. (**B**) SEM of uncoated and IonL-Phe-coated screws used as implants in this study before and after insertion into the edentulous alveolar crest. Arrows indicate areas where IonL aggregates on the screw surface.

A pilot study was conducted to evaluate the success rate, BIC%, and histopathological features utilizing IonL-Phe- and IonL-Met-coated implants in the same surgical procedure and analysis outlined in Wheelis et al. and to determine the appropriate sample size and formulation to assess multiple demographics using a paired model [62]. It was found that IonL-Phe (Figure 1A) demonstrated the best combination of success rate, BIC%, and histopathological observations, shown in Figure S1, compared with the uncoated control and IonL-Met, and therefore was chosen for this study.

### 2.2. Preparation of Implant and IonL-Phe Coating

Commercially pure titanium (cpTi) -threaded dentin pins (0.76 mm ⌀ × 2 mm, Fairfax Dental Inc., Miami, FL, USA) were used as implants in this study. All implants were cut to an approximate 2 mm length using orthodontic pliers and cleaned by sonicating for 45 min each in acetone, (DI) water, and ethanol solutions, respectively. After sonication, implants were placed in an oven at 60 °C to dry before being sterilized in an autoclave. 

Experimental implants receiving the IonL coating were dip-coated in a 50 mM ethanolic solution of IonL-Phe for 10 min to deposit approximately 0.5 μmol of IonL droplets on the screw surface. This concentration/amount of IonL-Phe was used as it demonstrated biocompatibility in a subcutaneous model [60]. After 10 min in the ethanolic IonL solution, implants were removed from the solution at a uniform rate of 60 µm/s with the assistance of a motorized stage (TA Instruments, New Castle, DE, USA) then placed in an oven at 60 ° C to dry for 48 h, then placed at room temperature for additional 24 h. Before surgery, coated and dried implants were placed under UV light for 1 h to maintain sample sterility before implantation. In order to evaluate the differences in implant surface and coating morphology before and after insertion in maxillary bones of rats in a non-survival surgery, scanning electron microscopy in high vacuum mode (SEM, JEOL SM-6010LA, Jeol, Peabody, MA, USA) was performed on 4 sets of 3 implants. Maxillary samples of euthanized 10–12-week old male Lewis rats (containing gingiva, bone, and blood) received 2 sets (1 coated and 1 uncoated) of screws. Immediately after implant placement, a circular bone saw was used to remove each implant without irrigation. This eliminated the potential disruption of the coating from additional wear, unscrewing, or release into the irrigation solution. The remaining two sets (1 coated, 1 uncoated) were characterized before insertion, with results shown in Figure 1B.

### 2.3. Animals 

All animal surgeries, as well as pre and postoperative care, were carried out with supervision and approval from the Institutional Animal Care and Use Committee (IACUC #16-05) in compliance with the NIH Guide for the Care and Use of Laboratory Animals. This study was divided into 4 experimental groups of Lewis rats (Charles River Laboratories, Wilmington, MA, USA) representing various demographic groups. These groups were chosen to evaluate the potential impact of the coating on healing due to biological variations in bone quality from age and gender. Details about the 4 animal demographic groups: young males, young females, old males, and ovariectomized (OVX) females are listed in Table 1. Young males and females were standard Lewis rats, while old males were retired breeders. OVX females (OVXF) received an ovariectomy at 12 weeks of age followed by 14 weeks of healing, similar to an established model performed by Du et al. [65]. Removal of ovaries creates conditions for estrogen deficiency-induced osteoporosis, similar to human menopause, resulting in a dysregulation of bone remodeling [65,66]. The OM group was chosen to simulate the detrimental effects of senescence on bone remodeling. Both OVX and OM allow the evaluation of any modulatory behavior of IonL in conditions where regenerative healing is impaired, as is sometimes the case in humans [30,66,67]. 

The rats were maintained in the Vivarium at the University of Texas at Dallas with sterile water and dry food pellets available to animals ad libitum, except for 72 h following surgery, in which the diet was crumbled and mixed with water. Experimental groups for histological evaluation were separated by time points (7 and 30 days, *n* = 10 per titanium treatment group, per demographic), while an additional experiment was performed on the young male group for molecular analysis. Experimental groups for molecular analysis were separated by time points at 2 and 7 days, with *n* = 6 per group with an additional 6 nonsurgical controls. A summary of the methods in this study, including the demographic groups, sample size, time points, and type of analysis, are summarized in Table 2.

### 2.4. Surgical Procedure

Rats were weighed before and after surgery to monitor body weight (Table 1). The animals were briefly anesthetized with isoflurane inhalation (1–4% mg/kg) and then given an intramuscular injection of 50–100 mg/kg ketamine hydrochloride with 20–50 mg/kg xylazine hydrochloride. After anesthesia, rats were placed in a dorsal decubitus position on a surgical table. Following positioning, animals were given an injection at the surgical site with 20 mg/kg of lidocaine with 1:100,000 epinephrine (Hospira Inc, Lake Forest, IL, USA) and 1.2 mg/kg sustained-release (SR) buprenorphine (ZooPharm, Windsor, US, USA) for analgesia. Implantation consisted of first making a 2 mm mucosal incision 1 mm in front of the right maxillary first molar to expose bone, followed by the drilling of a 0.67 mm implant bed using a surgical micromotor at 1000 RPM (NSK Surgic Pro) and subsequent placement of a 0.76 mm ⌀ by 2 mm titanium screw (coated or uncoated) in the edentulous alveolar crest osteotome site using needle holders (Figure 1). The procedure was then repeated on the contralateral (left) side of the maxillae, with the surgeon blinded to the side in which the coated or uncoated samples were placed. Additional control rats received no surgery (nonsurgical controls). Feeding, drinking, and grooming were monitored daily during the postoperative period. At the end of the experimental periods (Table 2), the animals were sacrificed with an overdose of pentobarbital sodium (Euthanasia III Med-Pharmex Inc., Pomona, CA, USA). After sacrifice, the implantation sites were cleaned briefly with saline before photos were taken using a stereomicroscope (Olympus, SMZ45T with DS-Fi2-L3 Camera, Shinjuku City, Tokyo, Japan) to track the clinical healing of the oral mucosa covering the implants. Experimental animals for histological analysis had their whole maxilla placed in 10% neutral buffered formalin (NBF). Experimental animals for molecular analysis had each tissue section containing either an implant or control tissue excised from the animal with dissecting scissors and then snap frozen. Additionally, three random femurs from rats of each demographic were collected and frozen at −20 °C in 1X phosphate-buffered saline (PBS) for further skeletal phenotyping by MicroCT analysis.

### 2.5. MicroCT Imaging and Analysis

Rat femurs from the various demographics were thawed and rehydrated in 1X PBS solution for 10 min before scanning and imaged using ultrahigh-resolution micro-CT imaging (OI/CT, MILabs, Utrecht, Netherlands) for additional skeletal phenotyping. Images were acquired at a voltage of 50 kV, a current of 0.21 mA, and an exposure time of 75 ms. Subsequently, projections were reconstructed using vendor software and converted to DICOM (Digital Imaging and Communications in Medicine) files using PMOD analysis software (PMOD Technologies LLC, Zurich, Switzerland) at a voxel size of 20 μm. Quantification of bone parameters was performed using Imalytics Preclinical (Gremse-IT GmbH, Aachen, Germany). Distal metaphysis of femur specimens was analyzed using a spherical region of interest (ROI) 3 mm in diameter, positioned in the trabecular area. The trabecular analysis included the acquisition of bone volume (BV, mm^3^), bone volume fraction (BV/TV %), trabecular thickness (Tb.Th, mm), and trabecular separation (Tb.Sp mm). BV (mm^3^) in cortical bone in the mid-diaphysis was analyzed using a cylindrical ROI 6 mm ⌀ by 2 mm in length.

### 2.6. Histological Processing 

Maxillae containing the implants were decalcified in 11.2% ethylenediaminetetraacetic acid (EDTA)-2Na at 4 °C for 2–3 weeks. After decalcification, whole maxillae were grossed down to transverse sections and separated into right and left sides containing the implant. After decalcification for an additional week and tissue processing with the implants in place, implants were carefully unscrewed from their coronal end before embedding in paraffin blocks. A total of 12 5 µm histological sections were made in the central region of the titanium implant site per biological replicate. Six sets of two serial sections were made with 30 μm of separation to get a good representation of the sample for staining.

### 2.7. Histopathological and Histolomorphometric Analysis

The progression of healing and osseointegration was evaluated using hematoxylin and eosin staining (H&E) (all demographics). One section at each depth (6 total) was used for histopathological evaluation and histomorphometry. Evaluation of soft tissue integrity and mucosa–implant interface consisted of the area from the alveolar bone crest to the point of implant emergence through the oral epithelium of the peri-implant mucosa. Evaluation of hard tissue integrity consisted of the area of implant threads in contact/adjacent to the alveolar bone, excluding any sample that penetrated the maxillary sinus. Soft tissue histomorphometry (YM 7 days only) was used to quantify blood clots, blood vessels, inflammatory cells, foreign body giant cells (FBGC), fibroblasts, and fibers in three 173.4 µm × 130.1 µm histological fields per section at 400× magnification, which were averaged. Bone-to-implant contact percentage (BIC%) was calculated on 30-day samples (all demographics) using Cellsens software (Olympus, Shinjuku City, Tokyo, Japan) to measure the length of the alveolar bone in direct contact with the implant. Before measuring the length of the implant at the bone level, a horizontal line designating the crest of the maxillary bone and crest of the maxillary sinus adjacent to the implant was drawn across the implant space of each section. Following this, several measurements were taken:

1. The entire length of the implant under the crest of the maxillary bone and above the crest of the maxillary sinus (Implant Length).
2. The length of the implant in contact with bone under the crest of the maxillary bone and above the maxillary sinus (Bone Contact).

BIC% was then calculated by using the equation:

BIC% = (Bone Contact)/(Implant Length) × 100(1)

Due to the size limitations of the model, the classic definition of osseointegration, direct bone-to-implant contact viewed histologically, was used. Implants with >60% direct bone-to-implant contact were considered successful based on human histological observations of clinically osseointegrated implants [68,69]. Hard tissue histomorphometry (YM only) was used to quantify blood clots, blood vessels, inflammatory cells, FBGC, fibroblasts, fibers, osteoblasts, osteoclasts, and new bone matrix in seven 173.4 µm × 130.1 µm histological fields per section at 400× magnification, which were averaged. 

### 2.8. Immunohistochemistry

Immunohistochemistry was used to identify different subpopulations of macrophages within threads adjacent to/in contact with oral mucosa and alveolar bone in the YM demographic. Macrophages/monocytes were identified using a universal macrophage marker CD68 (anti-CD68, 1:1000 Rabbit polyclonal (ab125212), Abcam, Cambridge, UK), while proinflammatory (M1) and anti-inflammatory (M2) subpopulations were identified by CD86 (anti-CD86, 1:2000 Rabbit polyclonal (PIPA 588284) Invitrogen, Carlsbad, CA, USA) and CD163 (anti-CD163, 1:500 Rabbit polyclonal (ab182422), Abcam, Cambridge, UK), respectively. Sections were first deparaffinized and then underwent antigen retrieval by submersion in Tris Buffer at pH 9.0 maintained at 95 °C for 30 min. After washing in DI water, the area of interest for staining was marked with a peroxidase-antiperoxidase (PAP) pen. Tissue was blocked with protein block provided from mouse and rabbit specific HRP/DAB (ABC) and Micropolymer Detection IHC Kit (Abcam, Cambridge, UK) and subsequently incubated with the selected primary antibody at 4 °C overnight in a humidified chamber. One section from each sample was stained with each marker, in addition to a final sample that was incubated with 1% bovine serum albumin in 1× phosphate-buffered saline (Sigma-Aldrich, St. Louis, MO, USA) instead of a primary antibody as a negative control. After incubation, the slides were washed and blocked with hydrogen peroxide before incubation with a goat anti-rabbit HRP Conjugate and 3,3′-diaminobenzidine (DAB) chromagen, following the manufacturer’s protocol from the Micropolymer Abcam IHC kit. Lastly, slides were counterstained in Mayer’s Hematoxylin for 2 min and finished with Permount (Fisher Scientific, Hampton, NH, USA) and a coverslip. Seven 173.4 µm × 130.1 µm histological fields were captured comprising the region adjacent to the implant at the alveolar bone level to quantify macrophages in the hard tissue. Three 173.4 µm × 130.1 µm histological fields were captured comprising the region from alveolar bone crest to the point of implant emergence through oral epithelium to quantify macrophages in the soft tissue. Cell counting was performed using the same technique employed with H&E-stained sections. As CD86 and CD68 are also markers for osteoclasts and some PMN granulocytes, these cells were identified based on their size, location, and nuclear morphology and excluded from analysis, so only macrophages were counted. 

### 2.9. Inflammatory Scoring

Inflammatory scoring was conducted in order to semi-quantitatively evaluate potential differences in acute and chronic inflammation from all the animal demographics. The scoring system (described in Table 3) was adapted from ISO 10993-6 to evaluate the number of neutrophils, macrophages, plasma cells, lymphocytes, and FBGC in soft and hard tissue adjacent to the implant [66]. Three 173.4 µm × 130.1 µm random histological fields were captured, comprising the region from both soft and hard tissue regions for scoring, with the score from the three regions from each tissue type being averaged.

### 2.10. Molecular Analysis

Fresh tissue sections comprising the peri-implant mucosa and bone were snap frozen and stored at −80° C to preserve RNA integrity in order to perform gene expression analysis. Approximately 50 mg of peri-implant or control tissue from each sample were homogenized using the Bullet Blender Storm (Next Advance Inc., Troy, NY, USA) according to the protocol outlined by Carter et al. 2012 [70]. RNA was isolated using the RNeasy Mini-kit (Qiagen, Hilden, Germany) following the manufacturer’s instructions. The concentration and quality of the RNA were verified with a spectrophotometer (NanoDrop™ 200, Fisher Scientific, Hampton, NH, USA) and a fragment analyzer (Agilent Technologies, Santa Clara, CA, USA). After isolation, cDNA synthesis was performed using qScript cDNA supermix (QuantaBio, Beverly, MA, USA), and cDNA reaction products were purified with the Qiaquick Purification Kit (Qiagen, Hilden, Germany). Real-time quantitative polymerase chain (RT-qPCR) was performed with cDNA and TaqMan gene expression assays (Applied Biosciences, Foster City, CA, USA) to quantify genes for macrophage polarization (*Arg1, Cd163, Nos2*), inflammation (*Ccr2, Ccr5, Cd80, Cxcl12, Il6, Il10, and Tnf*), tissue reconstruction and bone formation/remodeling (*Fgf1,Tgfb1, Vegfb, Col1a1, Cxcr4, Alpl, Bmp2, Bmp7, Ibsp, Dmp1, Bglap* (produces Osteocalcin-OCN), *Tnfrsf11b* ( produces Osteoprotegrin-OPG), Spp1 (produces Osteopontin-OPN), *Tnfsf11* (produces RANKL), Runx2, and Sost) using 10 ng/µL of cDNA per reaction. Each sample reaction was performed in triplicate on a QuantStudioTM 6 Real-Time PCR System (Applied Biosystems). Data analysis was performed using the ΔΔCt method to compare each marker of interest with 3 housekeeping genes (*B2m, Hprt1, Eif2b*), determining fold changes in IonL-coated and uncoated Ti samples relative to a nonsurgical control.

### 2.11. Statistical Analysis

All quantitative results were evaluated with the Shapiro–Wilk test to determine normality before statistical testing. Hard and soft histomorphometry, as well as IHC, underwent a two-tailed paired *t*-test if results distribution were normal or a Wilcoxon signed-rank test if not. Molecular analysis results were evaluated for outliers using the ROUTs test (Q = 1%) because of the nature of sample collection. After removal of outliers, molecular analysis results for each marker were evaluated with a two-tailed unpaired *t*-test if normal or a Mann–Whitney *U* test if not. Inflammatory scoring was evaluated using a two-way ANOVA to compare YF vs. YM, YM vs. OM, and OVXF vs. YF groups with only demographic and coating presence as factors. Tukey’s multiple comparisons were performed as a post hoc test after the ANOVA. BIC% was evaluated with a two-tailed paired *t*-test/Wilcoxon signed-rank test within each demographic and an equivalence test for the YM group. The tolerance range for equivalence was determined for BIC% from Wheelis et al. using the same model [62]. If the 90% confidence interval of BIC% from IonL-Phe-coated Ti was within the defined tolerance range of ±1 standard deviation of all samples BIC% from the model characterization, the coating was considered equivalent to uncoated Ti BIC%. Finally, a Yate’s Chi-square test was performed within each demographic group to determine if success rates were significantly different between coated and uncoated groups. All tests used a significance level (α) of 0.05. Table 2 summarizes the statistical testing performed for each method in this study.

## 3. Results

### 3.1. SEM and Clinical Analysis

**Figure 2:**
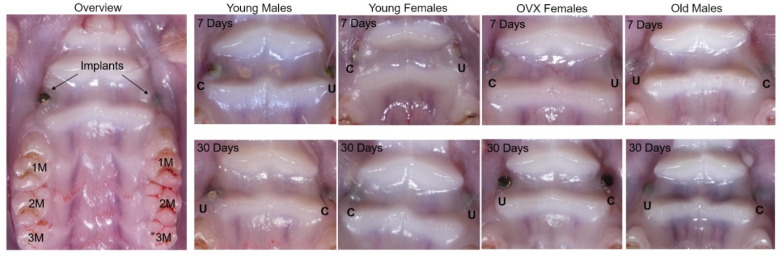
OM images of mucosal healing post implantation at 7 and 30 days in all demographic groups. Healing panel shows an overview of implant placement location relative to 1st, 2nd, and 3rd molar (1M, 2M, and 3M). Location of IonL-Phe-coated and uncoated implants are indicated by C and U, respectively.

SEM demonstrated the coating profile of IonL-Phe on the implants as well as the effect of insertion on coating stability. Prior to insertion, IonL-Phe formed aggregates concentrated on the root portions of the screw with some smaller droplets on the crest portions of the screw, shown in Figure 2. Following insertion and removal of screws in ex vivo maxillary surgeries, uncoated screws contained small amounts of bone and soft tissue debris. IonL-coated screws contained a larger amount of tissue debris compared with uncoated screws, particularly in areas where the IonL had aggregated. The IonL was no longer visible on the coated screw surface after removal but was likely still present and only obstructed by tissue debris. 

There were qualitative differences in bone quality observed during the surgical placement of implants into different animal demographics. YF bone required substantially less drilling time to create the implant bed than the YM group, while OVX female and OM required approximately 10 s of additional drilling time at 1000 RPM. Notably, there was surface-level fragmentation of bone directly adjacent to the implant bed of the OM group as the screws were placed, which was not observed in other demographics. After a sacrifice and sample collection, there were no clinical signs of infection among the four demographic groups following macroscopic evaluation, shown in Figure 2. All implants evaluated in the OVXF and OM groups remained in place for the duration of the study. One implant in the YM (2.5%, uncoated) group and three implants in the YF group (7.5%, IonL coated) lost primary stability before sacrifice at 30 days, and these implants were excluded from the histopathological and histomorphometric analysis. The YM and YF groups also had the largest weight changes during experimental time periods (Table 1). No implants were lost at the 7-day time point. 

### 3.2. Histopathological Analysis and Inflammatory Scoring 

**Figure 3:**
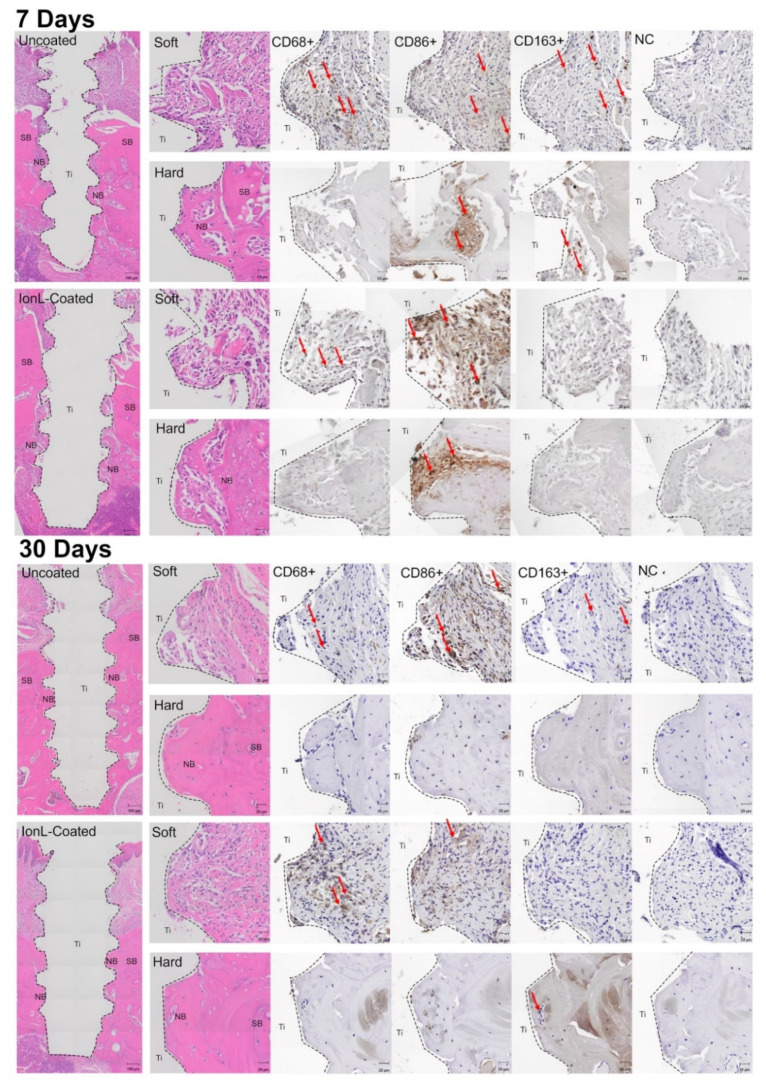
Histology representing peri-implant healing of uncoated and IonL-Phe-coated titanium over 7 and 30 days, H&E, and immunohistochemistry (IHC) for macrophages (CD68, CD163, CD86). Panel displays an overview of peri-implant tissue and a more detailed view of hard and soft tissue, respectively, from the same sample is all stains. NC is a negative control for IHC, SB is supporting bone, NB is new bone, and Ti is the void left by the implant after processing. Arrows indicate examples of positively stained cells for each marker.

H&E sections were analyzed with inflammatory scoring and qualitative histopathology to evaluate the effect of IonL coating on the progression of inflammation and healing at 7 and 30 days. In general, there was no evidence that IonL-Phe was still present in peri-implant tissues in all the demographics at these time points. YMs had similar healing and inflammatory progression in coated and uncoated samples at both 7 and 30 days. At 7 days, the peri-implant mucosa contained negligible residual blood clots from implantation and a mild inflammatory infiltrate consisting of predominantly mononuclear (MN) cells within maturing connective tissue (Figure 3). This peri-implant soft tissue was composed of fibroblasts, loose and disorganized collagen fibers, with several small capillary-sized vessels. Some samples contained sequestered bone fragments with one to two FBGC and dispersed neutrophils. The inflammation around these fragments was localized and did not affect the healing of adjacent hard or soft tissue. Tissue healing was advanced at the bone level at 7 days, with a negligible amount of blood clots and inflammatory cells. There was also a moderate to a large amount of osteoid formation surrounding implant threads at this time point. This osteoid appeared to be primarily woven cortical bone with some trabeculae adjacent to supporting bone. The osteoid also contained developing bone marrow space with fibroblast-like cells, new osteocytes being embedded in the recently formed bone matrix, and active osteoblasts lining the matrix. New osteocytes and osteoclasts were identified by their location, well-developed cytoplasm, and cuboidal shape. The location of this new bone apposition was identified by reversal lines following the same contour as Howship’s lacunae, suggesting there was osteoclast resorption activity on the edges of supporting bone along with empty osteocyte lacunae. 

**Figure 4:**
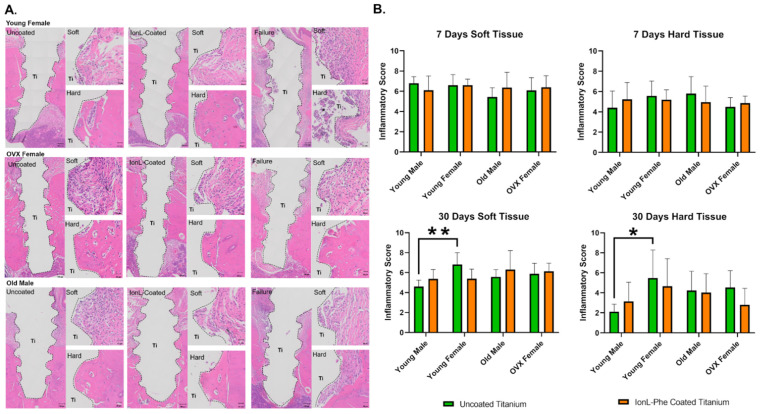
(**A**) Histology representing various characteristics of peri-implant healing in YF, OM, and OVXF groups, H&E panel displays the overview of peri-implant tissue in successful and failed samples, along with a more detailed view of hard and soft tissue, respectively. Ti is the void left by the implant after processing. * Indicates site of possible secondary infection. (**B**) Inflammatory scoring in all four demographic groups in IonL-Phe-coated and uncoated samples over time. * and ** indicate statistical significance among groups (* *p* < 0.05, ** *p* < 0.01).

At 30 days, there were no observable differences between healing in IonL-Phe-coated and uncoated Ti implants, shown in Figure 3. The YM group no longer had a blood clot in the oral mucosa, and inflammatory mononuclear (MN) cells were well dispersed within the tissue or at the Ti–tissue interface. The connective tissue in the oral mucosa was dense and mature, with fibers organized parallel to the implant axis. Some samples possessed a stratified and mature epithelial layer that was attached to the implant. Similar to the 7-day time point, there were also samples with residual bone fragments surrounded by FBGC and very localized inflammation/resorption. Qualitatively, blood vessel density was reduced at 30 days compared with 7 days. In the YM hard tissue, the implant space was surrounded by a new mature bone matrix with cortical structures containing several osteocyte lacunae and sparse haversian canals and trabecular structures that contained well-developed bone marrow. Inflammatory scoring in the YM group from 7 to 30 days indicated a slight decrease in the inflammatory infiltrate in both hard and soft tissue. IonL-Phe-coated samples had a slightly higher but nonsignificant (*p* > 0.05) score compared with uncoated Ti shown in Figure 4B. 

There were no large differences in histopathological features and inflammatory scoring between the YF, OVFX, and YM demographic groups at 7 days. However, the OM group demonstrated substantially less remodeling activity than the YM group at this time point. Differences in inflammation and healing between demographics became more evident at 30 days, shown in Figure 4A. The YF group was roughly half the bodyweight of the YM group, which consequently had less maxillary supporting bone for the implant (Figure 4). However, there were no significant differences in bone quality between YF and YM groups (Figure S2). Overall, the YF group had a persistent chronic inflammatory reaction containing both MN and PMN cells. Most samples contained dense PMN (neutrophils) clusters at the hard and soft tissue level indicating possible secondary infection in both coated and uncoated samples, with partial or full fibrous encapsulation. This was corroborated by inflammatory scoring, as YFs had significantly higher scores than the YM group in uncoated implants only (*p* < 0.05). A few YF samples without chronic inflammation healed similarly to the YM group, with normal bone matrix formation. 

In the OVXF group, soft tissue within the implant threads appeared disorganized with unremitting MN inflammatory cells. At 30 days, there was a varied bone response, with some samples showing a lack of remodeling activity, demonstrated by empty lacunae in supporting bone at this later time point. Other samples showed new bone formation into the threads, with various amounts of marginal bone loss on the coronal portion of the implant. Finally, the OM group had a similar amount of supporting bone to the YMs, but unlike the younger animals, the new bone matrix did not appear well-integrated into the supporting bone. OMs also possessed significantly higher metaphysis bone volume and lower trabecular separation than the YMs group (Figure S2), with marginal bone loss and lack of bone reactivity. 

Overall, the YMs did not exhibit any significant differences in inflammation or healing between coated and uncoated samples (Figure 4b). YFs and OMs groups had elevated inflammatory scores compared with the YMs group and qualitatively demonstrated reduced bone formation, fibrous encapsulation, and possible infection. 

### 3.3. BIC% and Success Rate

Bone-to-implant contact and the clinical success rate was calculated according to Equation (1) for all demographics at 30 days, and their values are displayed in Table 4. YMs achieved 58.53 ± 20.94% BIC in uncoated samples and 64.80 ± 8.466% BIC in coated samples. BIC and success rate were not significantly different between the coated and uncoated groups. As a result, an equivalence test was performed utilizing a tolerance range defined by the performance of uncoated Ti in Wheelis et al. [62]. IonL-coated samples were equivalent to uncoated samples. OVXFs achieved 40.89 ± 24.64% BIC in uncoated samples and 43.54 ± 26.12 BIC in coated samples, resulting in a success rate of 20% and 50%, respectively. Like the YM group, there was no statistically significant difference in BIC between coated and uncoated groups. However, according to clinical definitions of success (>60% BIC), the coated group in OVXF was determined to have a statistically significant increase in success/failure ratio compared with the uncoated OVXF group. 

The YFs achieved 17.85 ± 30.01% BIC in uncoated samples and 33.37 ± 36.86% BIC in coated samples, resulting in a success rate of 28.57% in both groups. Finally, the OMs achieved 21.88 ± 23.06% BIC in uncoated and 24.70 ± 12.72% BIC in coated samples, resulting in a success rate of 10% and 0%, respectively. Overall, an ANOVA (Table 4) determined biological variation from the differing demographics significantly affected BIC% (*p* < 0.001) while the IonL did not (*p* > 0.05).

**Table 4:** Bone-to-implant contact and success rate of all demographics at 30 days.

	Young Males (*n* = 9)		Young Females ^a^ (*n* = 7)		Old Males ^a^ (*n* = 10)		OVX Females (*n* = 10)	
	Uncoated Ti	IonL-Phe	Uncoated Ti	IonL-Phe	Uncoated Ti	IonL-Phe	Uncoated Ti	IonL-Phe
**BIC (%)**	58.53 ± 20.94	64.80 ± 8.466 ^d^	17.85 ± 30.01	33.37 ± 36.86	21.88 ± 23.06	24.70 ± 12.72	40.89 ± 24.64	43.54 ± 26.12
**Success Rate (>60% BIC)**	66.67%	77.78%	28.57%	28.57%	10.00%	0.00%	20.00%	50.00% *

* Indicates IonL group has a statistically significant difference in the distribution of success/failures in Yate’s Chi-Square test compared with the uncoated group (*p* < 0.05). ^a^ Indicates statistically significant difference in BIC from the YM group using two-way ANOVA (*p* < 0001). ^d^ Indicates the IonL-coated group is within the tolerance range defined by Wheelis et al. and is noninferior to the uncoated group.

### 3.4. Histomorphometric Analysis in Young Males

**Figure 5:**
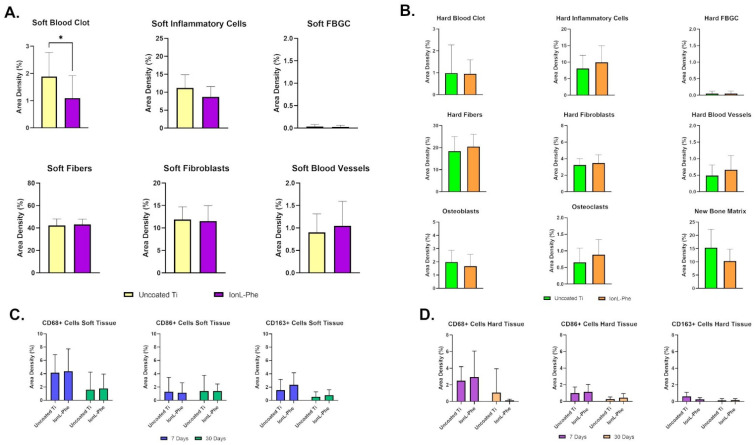
Histomorphometry of (**A**) soft tissue and (**B**) hard tissue healing parameters in YM group at 7 days. Morphometry of CD68, CD86, and CD163 positive cells (**C**) soft and (**D**) hard tissue of YM uncoated and IonL-Phe coated Ti at 7 days. (*n* = 10). * Indicates statistical significance among groups (*p* < 0.05).

Histomorphometry of all YM samples was conducted in order to quantitatively evaluate the difference in inflammation, tissue reconstruction, and bone remodeling between coated and uncoated samples at 7 days, shown in Figure 5A,B. In the soft tissue, blood clot present in IonL-Phe-coated Ti was significantly decreased compared with uncoated samples (*p* < 0.05). On the other hand, there was no difference in the number of inflammatory cells, fibers, fibroblasts, and negligible amounts of FBGC between both groups in soft tissue. IonL-Phe-coated samples had increased density in blood vessel formation compared with the uncoated group, but it was nonsignificant. The same trends were observed in the hard tissue for blood clots, FBGC, fibers, fibroblasts, and blood vessels. The density of inflammatory cells trended higher in IonL-Phe-coated samples compared with uncoated Ti. Among the bone remodeling cells and structures, IonL-Phe had decreased osteoblasts and new bone matrix but increased osteoclast density compared with uncoated Ti. However, none of these changes were considered significant.

Immunohistochemistry of YM sections at 7 and 30 days was conducted in order to evaluate the progression of healing through the presence of macrophages (CD68) and their respective proinflammatory (CD86) and anti-inflammatory (CD163) phenotypes. Histopathological observations indicated that CD68+ and CD86+ cells appeared highest in density in both soft and hard tissue at both time points and generally were present throughout the ingrowth of new tissue into the implant threads (Figure 3). CD163+ cells appeared lower in density and were present closer to the supporting tissue rather than at the Ti surface. The density of macrophages appeared similar in coated and uncoated samples. Morphometry of both groups helped confirm these histopathological observations, shown in Figure 5C,D. Counting indicated that CD68+ cells were highest in density at 7 days in the soft tissue, while the proportions of CD86+ and CD163+ cells were roughly equal in density, shown in Figure 5C. From 7 to 30 days, the density of CD68+ and CD163+ cells decreased while CD86+ cell density was similar to the 7-day density. IonL-Phe coated samples had higher CD68+ and CD163+ cell densities at 7 and 30 days, but they were nonsignificant. The overall density of macrophages in the hard tissue (Figure 5D) was lower than the density in the soft tissue at both time points for both groups. In the hard tissue, there were similar trends as those observed within the soft tissue. CD68+ cells were the highest in density, followed by CD86+ and CD163+ cells in decreasing order. From 7 to 30 days, macrophage density decreased in both groups across all markers. IonL-Phe samples had an increased density of CD68+ and CD86+ cells at 7 days in hard tissue, but not significantly. Overall, there was no significant difference in the expression and temporal behavior of macrophages between coated and uncoated titanium.

### 3.5. Molecular Analysis in Young Males

**Figure 6:**
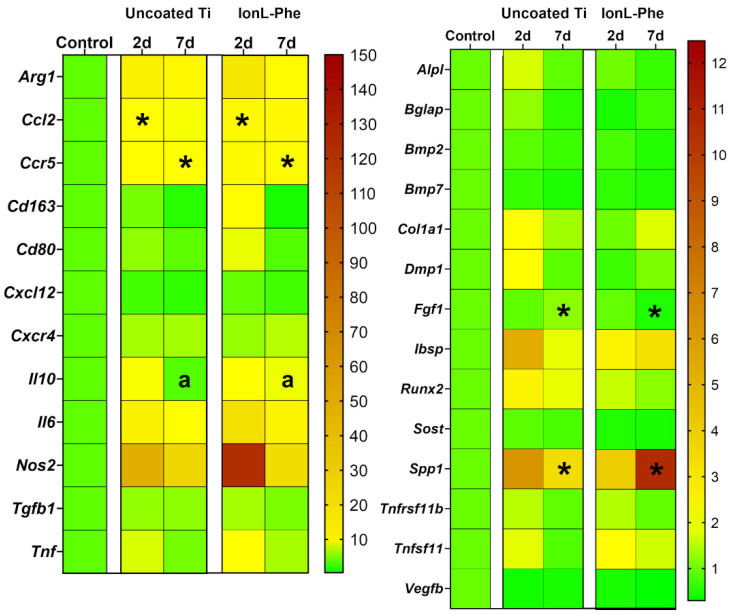
Heat map displaying the fold change of inflammatory, tissue reconstruction, and bone remodeling markers in peri-implant tissue around IonL-coated and uncoated titanium implants over time relative to nonsurgical control (*n* = 5). * indicates statistical significance among groups within that time point (* *p* < 0.05). **^a^** indicates *p* = 0.052 within that time point.

Gene expression analysis was conducted in order to evaluate the effect of IonL-Phe on inflammation and osseointegration at the molecular level when the coating is still present on the surface. Proinflammatory markers *Nos2, Cd80, Tnfα,* and *Il6* all had higher fold change in expression at 2 days in IonL-Phe-coated samples, although this elevation was not significant (shown in Figure 6 and Figure S3). Anti-inflammatory markers *Arg1, Cd163,* and *Il10* were also higher in fold change than uncoated samples at 2 days. Chemoattractant markers *Ccl2, Ccr5, Cxcr4, Cxcl12,* and *Tgfβ* also all had higher fold changes in expression at 2 days in IonL-Phe samples, although only *Ccl2* showed a significant increase (*p* < 0.05). By 7 days, most of these elevated fold changes had decreased back to the levels expressed in the uncoated samples, except for *Ccl2, Ccr5, Tnfα, Cxcr4,* and *Il10*. All of these markers still had elevated expression levels in IonL samples compared with the uncoated Ti, especially *Ccr5*, which was significantly upregulated (*p* < 0.05). In terms of tissue reconstruction and bone remodeling, most osteoblast and bone matrix markers were downregulated in IonL-Phe samples compared with the uncoated Ti, while more general markers *Fgf1*, and *Vegfβ* were unchanged between groups at 2 days. The only bone remodeling marker with higher fold change expression at 2 days in IonL-Phe samples was *Tnfsf11*. However, none of the changes in IonL-Phe samples were significantly different from the expression of these markers in uncoated samples. Interestingly, at 7 days, the trend was reversed for *Col1α1, Dmp1, Ibsp, Spp1,* and *Tnfsf11*, all being higher in expression in IonL samples, with *Spp1* being significantly higher (*p* < 0.05). *Fgf1* was also significantly downregulated in IonL samples compared with the uncoated Ti at 7 days (*p* < 0.01). The remaining bone markers *Alpl, Bglap, Runx2, Sost, Tnfrsf11b, Bmp2,* and *Bmp7* either showed no change or a decreasing trend in expression in IonL-Phe samples compared with the control. 

## 4. Discussion

Recent interventions aimed at modulating dental implant surfaces to improve success outcomes have focused on either addressing mitigating factors or encouraging regenerative healing [35]. Regardless, there is still the need for a multifunctional approach in Ti that can address various mitigating factors while encouraging regenerative healing in nonideal conditions. IonLs have been previously evaluated in vitro for proposed multifunctionality [51,57,59,71] and subcutaneously for biocompatibility in vivo [60]. Still, it was essential to consider their impact on oral osseointegration, as it represents a substantially more complex healing scenario [60]. Therefore, the aim of this study was to evaluate the effect that IonLs have on early healing and osseointegration of titanium implants for the first time. It was hypothesized that the IonL-Phe coating would maintain normal Ti–tissue interactions in several animal demographics and therefore be an excellent candidate for applications where mitigating factors are more commonly present, such as peri-implantitis. 

In ideal implant healing conditions, surgical placement causes damage to the surrounding tissue. This damage releases molecular signals necessary for initiating acute inflammation [18,21]. This stage of healing appears at 2 days in this model and is identified by the presence of blood clots and inflammatory infiltrate consisting of both PMN and MN cells [62]. Progression of acute inflammation is essential to determining success outcomes and was especially relevant to evaluating the IonL coating, as it has been demonstrated that it is still on the surface of Ti after 2 days in vivo [60]. At 2 days, uncoated Ti demonstrated the same gene expression profile as defined in Wheelis et al. (Figure 6.) [62]. While IonL-Phe-coated samples demonstrated an increase in pro and anti-inflammatory markers associated with chemotaxis (*Ccl2, Ccr5*) [72,73] and differentiation of monocytes into M1 and M2 macrophages (*Cd80, Nos2, Tnf, Il10, Arg1, Cd163*) [26,74,75,76,77,78,79], suggesting there was an increased acute inflammation in the presence of the coating (Figure 6). Conversely, there was a downregulation of osteoblast differentiation markers *Alpl* and *Runx2*, along with bone matrix markers *Bglap, Ibsp, Col1a1, Dmp1* [26,80,81,82], and remodeling markers *Spp1, Tnfsf11*, and *Tnsf11b* [83,84,85] in coated versus uncoated samples. It is important to note that *Alpl, Col1a1, Runx2, Ibsp, Spp1, Tnfsf11,* and *Tnfrsf11b* were still upregulated in coated samples relative to a nonsurgical control, so bone remodeling is still occurring in coated samples, just not to the same magnitude as uncoated Ti at 2 days. Therefore, increased acute inflammation from IonL-Phe may account for the trend towards a slightly delayed onset of bone remodeling earlier in healing. There has been an extensive evaluation of the positive correlation between IonL structure hydrophobicity and toxicity toward mammalian and bacteria cells [51,59]. Although IonL-Phe was employed in a concentration known to maintain antimicrobial activity and mammalian cell compatibility, the structures’ hydrophobicity (Figure 1A) could still result in some degree of cellular apoptosis via surfactant toxicity [51,86]. This damage occurred in addition to damage associated with implant placement. This could have resulted in increased release of DAMPs and therefore increased recruitment of cells associated with innate immunity [20,21]. However, more exploration into the proteins present on the surface of the coated implants at this time is needed to confirm this hypothesis. Interestingly, coated samples also demonstrated the upregulation of genes for anti-inflammatory cytokines *Arg1, Cd163,* and *Il10* [21], regulating acute inflammation. The coating behavior of IonL leaves most of the Ti surface exposed (Figure 1B), which likely allowed for direct Ti–protein interactions, maintaining desired healing progression.

It is important to re-emphasize that surfactant toxicity is a key component of the IonLs’ antimicrobial activity, allowing for some control over the microenvironment [59]. IonL-Phe is also observed to release 40% of its original concentration from the surface of Ti in PBS within 7 days in vitro, so any mammalian cell cytotoxicity is temporary and likely accelerated in vivo by the phagocytosis of neutrophils and macrophages [51]. Accordingly, at 7 days, there was no indication that the IonL was still in surrounding maxillary tissue. This temporal release behavior is thought to prevent interference with inflammatory resolution and initiation of bone remodeling [62]. RT-qPCR supported this observation, as inflammatory markers in coated samples were decreased back to the uncoated levels, except for *Ccr5, Il10*, and *Cxcr4* (Figure 6 and Figure S3). These markers are associated with macrophage, lymphocyte, and MSC recruitment, as well as regulation of bone remodeling [26,87,88]. Additionally, *Col1α1, Ibsp*, and *Tnfsf11* were expressed higher at 7 days compared with uncoated Ti. This suggests that tissue regeneration in the IonL-coated samples was still ongoing—a possible compensation for more intense acute inflammation at 2 days. These trends in gene expression were supported with histological observations at 7 days showing normal healing progression in both sample groups (Figure 3). Inflammatory scoring, histomorphometry, and IHC for the YM group suggest that inflammation may be slightly elevated in coated samples (Figure 3, Figure 4 and Figure 5) but not significantly. 

From 7 to 30 days in this rodent model, bone remodeling progressed in the absence of acute or chronic inflammation, resulting in successful osseointegration [62]. The healing progression of coated and uncoated samples was the most similar at 30 days clinically and histopathologically, with negligible inflammation and healthy new mucosal and bone tissue (Figure 2, Figure 3 and Figure 4). The success rate for coated samples was approximately 11% higher and less variable (coated BIC had approximately 60% lower standard deviation) than uncoated samples (Table 4). While this improvement is not statistically significant, it was a trend worth investigating. This model was designed to evaluate the coatings’ impact on ideal conditions for osseointegration; however, we do know that 20–30% of the implants placed in this model will fail due to fibrosis [62]. Some failures exhibited only MN inflammation (stability), while others had a potential infection due to an accumulation of neutrophils [62]. Failures in this study possessed similar features; however, failed coated samples did not exhibit signs of secondary infection in YMs. This suggests that IonL-Phe could create conditions early in healing to prevent bacterial infiltration.

Investigating additional demographic groups allowed us to evaluate the impact that biological factors have on IonL performance and normal Ti healing. YFs possessed dense PMN inflammation at 30 days in both sample groups, suggesting a secondary infection (Figure 4). This was likely due to issues with implant stability related to the demographic’s size and growth over the experimental period (Table 1). This instability likely created pockets for biofilm growth around the implant, which resulted in a major increase in the microbial challenge [89,90,91]. Although the IonL is antimicrobial, it was released from the surface before 7 days, so it is, therefore, unable to overcome this bacterial challenge [59]. Still, implant instability is likely the primary cause of failure, and when the samples in this group do osseointegrate successfully, they appear similar to the YM group. Micro-CT (Figure S2) also indicated that the quality of bone is optimal in YF, but there is not enough bone to stabilize the implant. Fortunately, the application of IonL did not have any detrimental effect on BIC% or inflammatory score in YFs, as there was no statistical difference between coated and uncoated groups. 

Micro CT (Figure S2) indicated that there was reduced bone quality in the OM group compared with the YM group. Based on histopathological observations, failed implants were likely a result of estrogen deficiency-induced osteopenia or osteoporosis. Evidence from preclinical studies has concluded that overall bone quality is reduced by estrogen deficiency [92]. This reduction is caused by decreased TGFβ1 and OPG production, which delays normal MSC and osteoblast activity, with a simultaneous increase in osteoclastogenesis markers, RANKL, and IL6 [66,83,92,93]. This dysregulation causes impaired healing, lower BV, and trabecular thickness, as observed in this study (Figures S2 and S4) [66,67,92,94]. It is possible that the increase in acute inflammation caused by the coating counteracted some of the MSC recruitment delays, but the more in-depth analysis is needed to explore this phenomenon. Clinical, microtomographic, and histological observations (Figure 2, Figures S2 and S4) suggested OMs may have had a higher cortical bone mineral density than the YM Group. Aged rodents typically have more brittle cortical bones due to a lack of remodeling activity and are likely immunosenescent [67]. Immunosenescence arises during aging and is associated with dysregulation of the innate immune system, caused by alterations in macrophage density, polarization, and function [95,96]. Unlike OVXF, which still exhibited more new bone deposition and remodeling, these OMs have been impaired at an even earlier stage in healing, resulting in even less BIC than OVFXs. Still, BIC% increased by 2.82 % in coated samples versus uncoated samples. 

This study has confirmed that regardless of detrimental biological factors, IonL-Phe does not negatively affect titanium osseointegration in ideal conditions. Still, the limitations of the study need to be discussed. Healing parameters were well defined for this model in YM [62]. However, the overall healing behavior of the YF, OVFX, and OM groups in this model demonstrated the negative effects that biological and anatomical variation can have on model success. For example, stability issues in the YF group could have been prevented by decreasing implant size. Additionally, the mechanisms by which the IonL influenced the initial protein layer formation and, therefore, subsequent healing events need to be elucidated. Understanding these mechanisms would allow further functionalization or applications in more complex healing environments, i.e., immunocompromised or diabetic patients. Regardless, IonL-Phe is an excellent candidate for further assessment in scenarios known to be challenged by bacteria, such as in patients with a history of periodontal disease, or to improve re-osseointegration after peri-implantitis treatment.

## 5. Conclusions

Taking into consideration all metrics, IonL-Phe-coated Ti induced an increased acute inflammatory response at 2 days that was resolved similarly to uncoated Ti, as the IonL was resorbed/released from surrounding tissues by 7 days. By 30 days, it was evident that the coating had no detrimental effect on soft tissue and bone integration, BIC% or success rate in both ideal (YM) and nonideal (YF, OVF, OM) conditions where stability or bone quality may be compromised. Therefore, IonL-Phe is a biocompatible implant coating. IonLs may potentially address a clinical need for surface treatment approaches for titanium implants that can effectively mitigate conditions that lead to destructive inflammation and failure of dental implants without contributing to a destructive scenario. IonL-Phe is also an excellent template for the development of a new generation of implant coatings with multifunctional, regenerative, and functionalization capabilities. Future studies could evaluate the effectiveness of the coating in rescuing implants inflicted with peri-implant disease and explore the possibility of functionalizing the coating with immunomodulatory proteins, such as DAMPs, to address specific patient factors that impair or dysregulate healing. 

## Figures and Tables

**Table 1:** Demographic details of Lewis rats used in this study.

Demographic Group	Age Range (Weeks)	Average Weight at Surgery (g)	Average Weight at Sacrifice (g)	Average Weight Change
Young Male (YM)	10–12	295.82 ± 39.80	354.64 ± 53.48	19.88%
Young Female (OF)	10–12	180.33 ± 10.60	206.71 ± 16.01	9.180%
Old Males (OM)	52–78	529.87 ± 45.03	524.95 ± 45.57	−0.9257%
Ovariectomized Females (OVXF)	26–28	290.55 ± 11.42	305.2 ± 16.85	4.378%

**Table 2:** Summary of methods used in this study.

Type of Analysis	Outcome	Groups	Healing Period	Result	Statistical Test
**Clinical Evaluation**	Overall health evaluation and resolution of inflammation at implant site	Young Males, Young Females, OVX Females, and Old Males	7 and 30 days	Figure 2, Table 1.	N/A
**X-ray Microtomography**	Supplemental evaluation of bone quality in femur.	Young Males, Young Females, OVX Females, and Old Males	N/A	Figure S2.	One-Way ANOVA with Tukey’s multiple comparisons test (YM vs. YF, YF vs OVXF, and YM vs. OM only)
**Histology (H&E)**	Qualitative progression of inflammation, wound healing, and osseointegration	Young Males, Young Females, OVX Females, and Old Males	7 and 30 days	Figure 3 and Figure 4.	N/A
**Histomorphometry** **(H&E-BIC%)**	Implant success rate	Young Males, Young Females, OVX Females, and Old Males	30 days	Table 4.	Yate’s Chi-Square–Success Rate (YM vs YF, YF vs OVXF, and YM vs. OM only)
					Two-Way ANOVA with Tukey’s multiple comparisons test (Demographic and Coating as factors, YM vs. YF, YF vs. OVXF, and YM vs. OM only)
					Paired *t*-test or Wilcoxon signed-rank test (Two-tailed, YM only) -BIC%
					Equivalence (YM only) -BIC%
**Cell Histomorphometry** **(H&E)**	Quantitative progression of inflammation, wound healing, and osseointegration	Young Males	7 days	Figure 5.	Paired *t*-test or Wilcoxon signed-rank test (Two-tailed)
**Inflammatory Scoring** **(H&E)**	Semiquantitative measure of degree of inflammation	Young Males, Young Females, OVX Females, and Old Males	7 and 30 days	Table 3, Figure 4.	Two-Way ANOVA with Tukey’s multiple comparisons test (Demographic and Coating as factors)
**IHC**	CD68 (Pan-Macrophage)	Young Males	7 and 30 days	Figure 5.	Paired *t*-test or Wilcoxon signed-rank test (Two-tailed)
	CD86 (M1 Macrophages)				
	CD163 (M2 Macrophage)				
**RT-qPCR**	Fold change in genes associated with inflammation, wound healing, and osseointegration	Young Males	2 and 7 Days	Figure 6.	Unpaired *t*-test or Mann–Whitney *U* test (Two-tailed)

**Table 3:** Scoring Method for Inflammatory Cells.

	Score				
Cell Type	0	1	2	3	4
Polymorphonuclear Cells (PMNs)	0	1–5 cells/per field (pf) ^1^	5–10 cells/pf	Heavy Infiltrate (>10 cells/pf)	Densely Packed
Macrophages	0	1–5 cells/pf	5–10 cells/pf	Heavy Infiltrate (>10 cells/pf)	Densely Packed
Lymphocytes	0	1–5 cells/pf	5–10 cells/pf	Heavy Infiltrate (>10 cells/pf)	Densely Packed
Plasma Cells	0	1–5 cells/pf	5–10 cells/pf	Heavy Infiltrate (>10 cells/pf)	Densely Packed
Foreign Body Giant Cells	0	1–2 cells/pf	3–5 cells/pf	Heavy Infiltrate (>5 cells/pf)	Densely Packed

^1^ Each field is 173.4 μm × 130.1 μm.

## Data Availability

All raw data are available to share upon request.

## Supplementary Materials

The following supporting information can be downloaded at: https://www.mdpi.com/article/10.3390/genes13040642/s1, Figure S1: OM and H&E images of healing postimplantation at 30 days in IonL-Phe and Met Implants.; Figure S2: X-ray Microtomography of Lewis rat femurs in different demographics.; Figure S3: Scatter plot displaying gene expression of peri-implant tissue in IonL-coated and uncoated titanium implants over time.
