# Supplementary material for: Effects of Dicationic Imidazolium-Based Ionic Liquid Coatings on Oral Osseointegration of Titanium Implants: A Biocompatibility Study in Multiple Rat Demographics
Source: Genes (Basel). 2022 Apr 2;13(4):642. doi: 10.3390/genes13040642 (PMC9026960; doi:10.3390/genes13040642)
Supplement: Supplementary file 1 [file genes-13-00642-s001.zip › IonL-Demo_Supporting Information for Publication_Final_Genes_R1.pdf]

Supporting Information for Publication

Effects of dicationic imidazolium-based ionic liquids on oral osseointegration of titanium implants: A biocompatibility study in multiple rat demographics.

Sutton Elizabeth Wheelis[1], Claudia C. Biguetti[1], Shruti Natarajan[2,3], Bhuvana Lakkasetter Chandrashekar[1], Alexandra Arteaga[1], Jihad El Allami[1], Gustavo Garlet[4], Danieli C. Rodrigues[1*].

Supporting Information Contains 6 Pages with 1 Equation and 3 Figures.

S1. IonL Power Analysis Study

Introduction

Ionic liquids 1,10-bis(3-methylimidazolium-1-yl) decane diphenylalanine (IonL-Phe) and 1,10-bis(3-methylimidazolium-1-yl) decane dimethionine (IonL-Met) have previously demonstrated biocompatibility on titanium (Ti) surfaces in a rat subcutaneous model[1]. However, the impacts of the coatings on oral osseointegration had not been investigated. The purpose of this study was to select the best formulation of IonL and sample size to use in the multiple demographic animal study. It was hypothesized that IonL-Met would be the most suitable for this study as its impact on the inflammation and healing response in the subcutaneous model was the most similar to the uncoated Ti control.

Materials and Methods

Medium doses of IonL- Phe (0.5 µmol) and IonL-Met (1.0 µmol) coated Ti were implanted into 10–12-week-old male Lewis rats to evaluate BIC and success rate (n=6, for each coating for a total of 12 rats). Synthesis and coating procedure is the same as outlined in section 2.1 and 2.2, only a 10 mM ethanolic solution was used to deposit approximately 1.0 µmol of IonL-Met on the surface. Surgical procedure was the same as outlined in section 2.4, except IonL coated samples were placed on the left sides of the maxilla, with a sham on the contralateral side to exactly replicate the previous pre-clinical model[2]. Rats were sacrificed at 30 days, and tissue was collected and processed in the same manner as section 2.6. Success rate and BIC% were calculated for the coated samples to determine the most suitable coating for evaluation in the main article. After selection of the coating, the difference between IonL-coated and uncoated sample BIC was used as minimum detectible difference, δ to calculate the sample size needed to confirm that difference with a two-sided two-sample t-test with 80 % power in the equation shown below.

$$n \geq \frac{2s^2p}{\delta^2}((t_{\alpha,v}) + (t_{\beta(1),v})^2$$

Equation S1. Sample size estimation for a t-test. n is sample size, s^2p is within population variability, δ is minimum detectable difference, $t_{\alpha,v}$ is the t critical value at $v = 2(n-1)$ degrees of freedom for a one sided t-test with $\alpha = 0.05$, and $t_{\beta(1),v}$ is the t critical value at v with $\beta = 0.20$.

Results and Discussion

It was found that IonL-Phe and IonL-Met coated samples achieved 100% and 83.3% success, respectively. Successful samples exhibited the same healing benchmarks as uncoated Ti for at least 30 days, shown in Figure S1. The failed IonL-Met implant exhibited inflamed soft tissue on the apical portion of the implant with dense neutrophils suggesting a secondary infection with partial fibrous encapsulation at the hard tissue level (Figure S1). Both IonL-Phe and IonL-Met have demonstrated antimicrobial activity *in vitro,* however IonL-Met coated Ti was less effective in reducing bacteria growth *in vitro* than IonL-Phe coated Ti[3,4]. This was likely due to a previously observed solidification behavior seen *in vitro* in saliva compounds. This solidification resulted in inactivation of antimicrobial activity, which may indicate that secondary infections are more likely with IonL-Met than IonL-Phe[3,4]. Therefore IonL-Phe was a better candidate for the larger animal study. Compared to the successful uncoated samples, BIC% decreased by 10.57% in IonL-Phe coated samples[2]. Using this difference, n=10 was calculated for 80% power in a two-sample one-sided paired t-test.

Conclusion

Due to a combination of observations from *in vitro* studies and this *in vivo* study, IonL-Phe has the potential to maintain biocompatibility of Ti while still maintaining proposed multifunctionality.

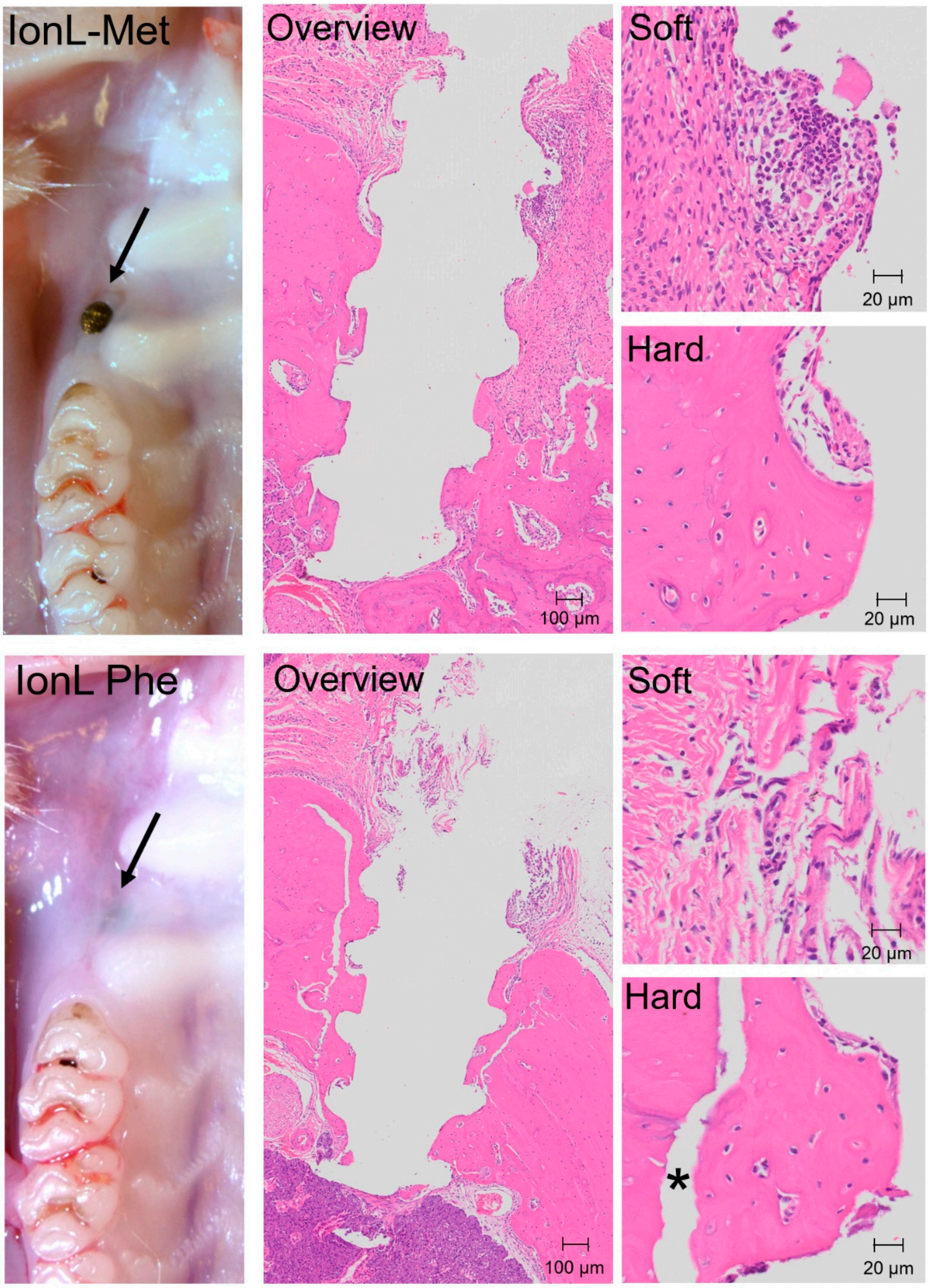

Figure S1. OM images (Left) of mucosal healing post-implantation at 30 days in IonL-Phe and Met Implants. Location of implants are indicated by arrows. Histology (middle and right) representing a peri-implant healing of IonL-Met and IonL-Phe coated titanium at 30 days). Panel displays overview of peri-implant tissue, and more detailed view of hard and soft tissue in H&E. * Indicated histological artefacts from sectioning.

S2. Demographic Micro CT

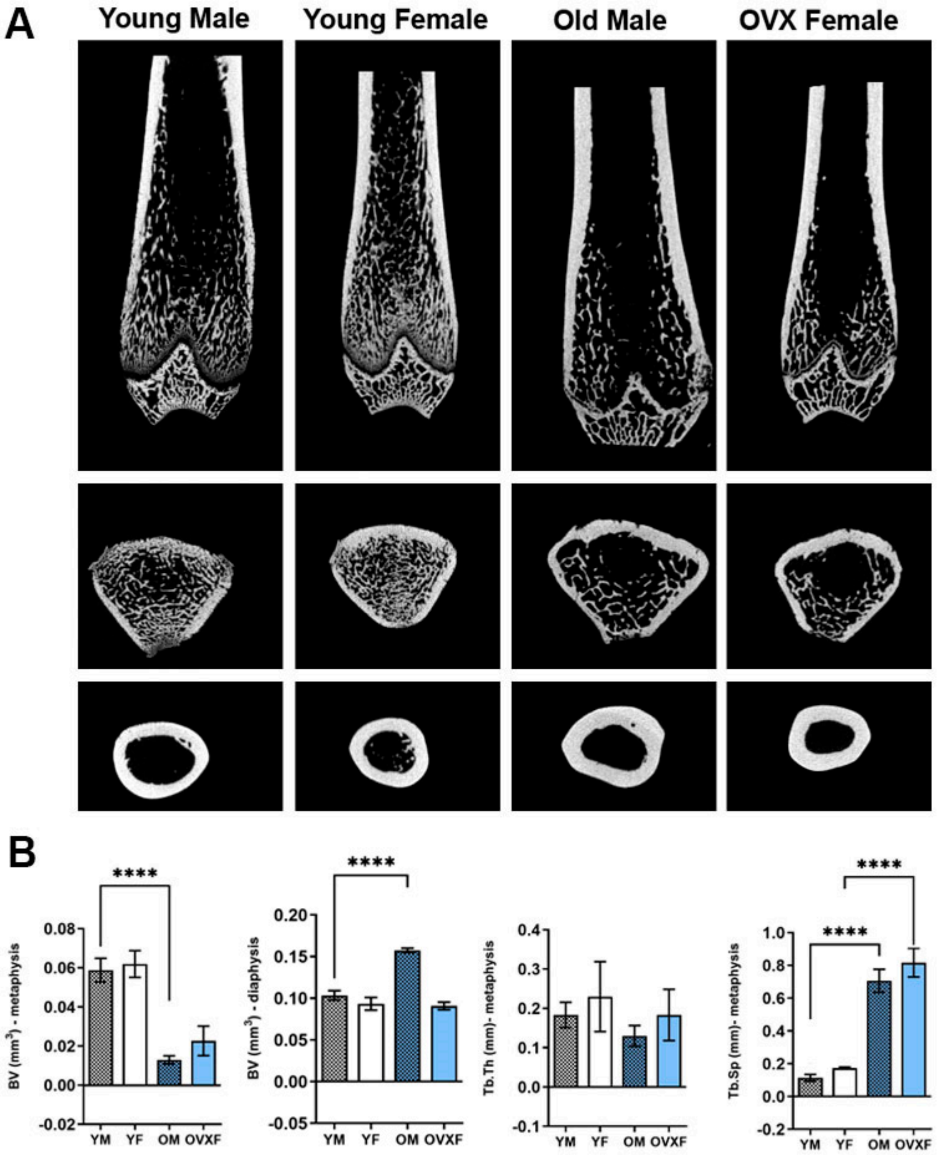

Figure S2. X-ray Microtomography of Lewis rat femurs in different demographics. A. Radiographs show the femur in the coronal (top), and metaphysis/diaphysis in the transverse plane (middle/bottom). B. Graphs indicating measurements for bone volume in the metaphysis and diaphysis and trabecular thickness and separation in the metaphysis. **** Indicates statistical significance among groups ($p < 0.0001$).

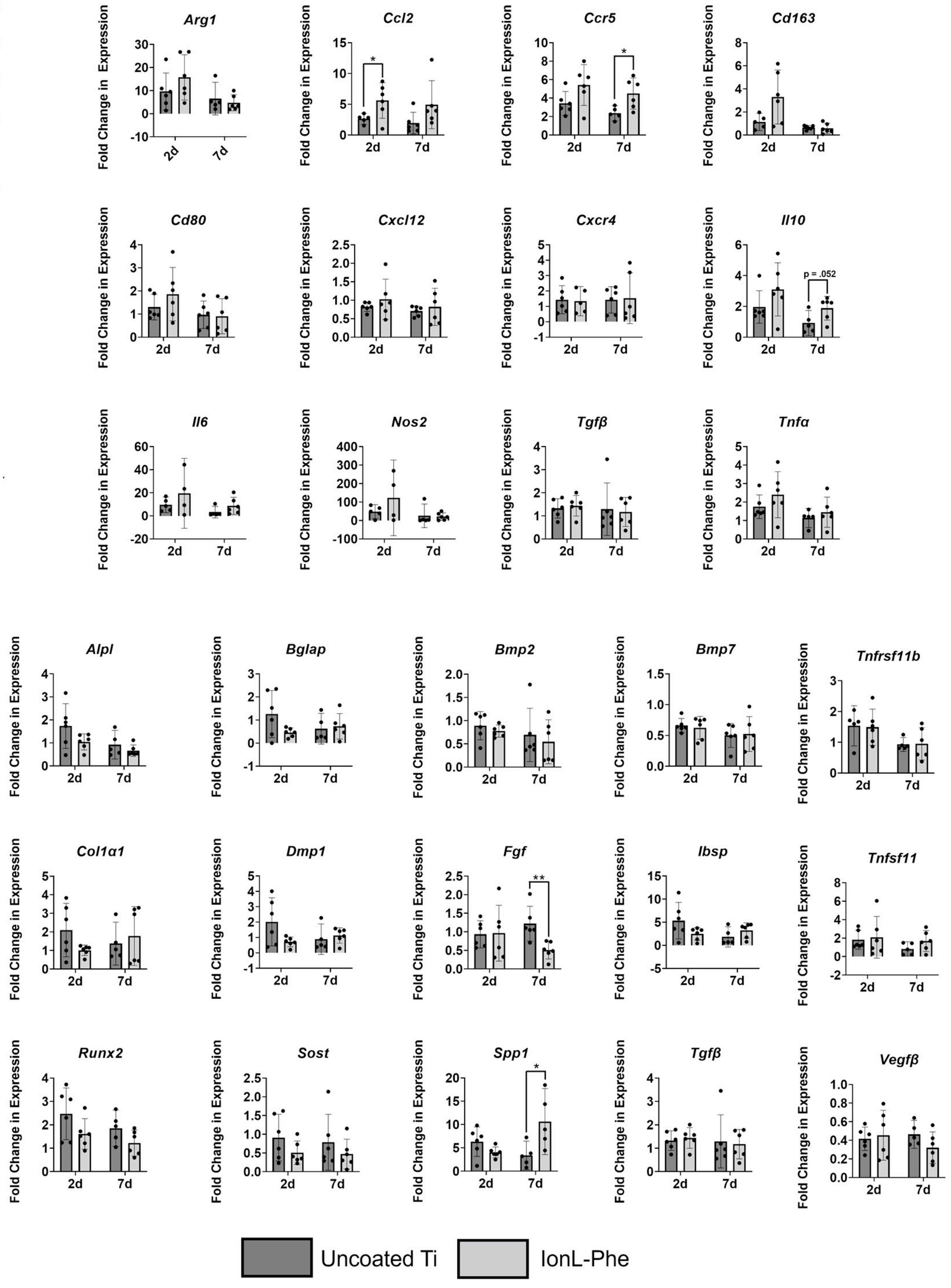

Figure S3. Scatter plot displaying gene expression of peri-implant tissue in IonL-coated and uncoated titanium implants over time with 95% CI (n=5), * and ** indicate statistical significance among groups (* p < 0.05, ** p<0.01).

References

(1) Wheelis, S. E.; Biguetti, C. C.; Natarajan, S.; Guida, L.; Hedden, B.; Garlet, G. P.; Rodrigues, D. C. Investigation of the Early Healing Response to Dicationic Imidazolium-Based Ionic Liquids: A Biocompatible Coating for Titanium Implants. *ACS Biomater. Sci. Eng.* **2020**, *6* (2), 984–994. https://doi.org/10.1021/acsbiomaterials.9b01884.

(2) Wheelis, S. E.; Biguetti, C. C.; Natarajan, S.; Arteaga, A.; el Allami, J.; Lakkasettar Chandrashekar, B.; Garlet, G. P.; Rodrigues, D. C. Cellular and Molecular Dynamics during Early Oral Osseointegration: A Comprehensive Characterization in the Lewis Rat. *ACS Biomater. Sci. Eng.* **2021**, *7* (6), 2392–2407. https://doi.org/10.1021/acsbiomaterials.0c01420.

(3) Gindri, I. M.; Palmer, K. L.; Siddiqui, D. A.; Aghyarian, S.; Frizzo, C. P.; Martins, M. A. P.; Rodrigues, D. C. Evaluation of Mammalian and Bacterial Cell Activity on Titanium Surface Coated with Dicationic Imidazolium-Based Ionic Liquids. *RSC Adv.* **2016**, *6* (43), 36475–36483. https://doi.org/10.1039/C6RA01003B.

(4) Gindri, I. M.; Siddiqui, D. A.; Bhardwaj, P.; Rodriguez, L. C.; Palmer, K. L.; Frizzo, C. P.; Martins, M. A. P.; Rodrigues, D. C. Dicationic Imidazolium-Based Ionic Liquids: A New Strategy for Non-Toxic and Antimicrobial Materials. *RSC Adv.* **2014**, *4* (107), 62594–62602. https://doi.org/10.1039/C4RA09906K.
