# Supplementary figures and images for: Effects of Dicationic Imidazolium-Based Ionic Liquid Coatings on Oral Osseointegration of Titanium Implants: A Biocompatibility Study in Multiple Rat Demographics
Source: Genes (Basel). 2022 Apr 2;13(4):642. doi: 10.3390/genes13040642 (PMC9026960; doi:10.3390/genes13040642)

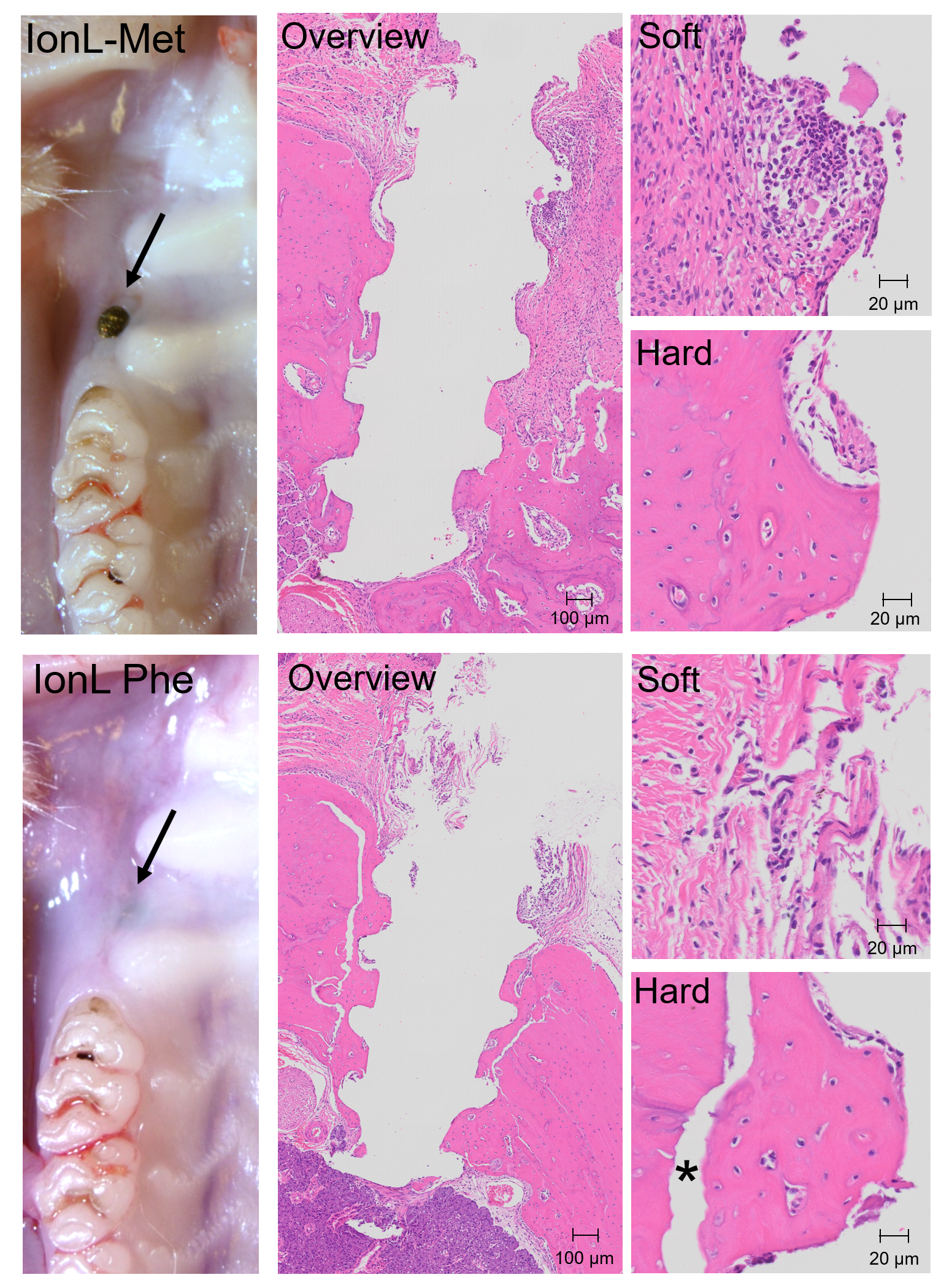

Supplement: Supplementary file 1 [file genes-13-00642-s001.zip › FigureS1_IonLMet&Phe_ACS_Final.tif]

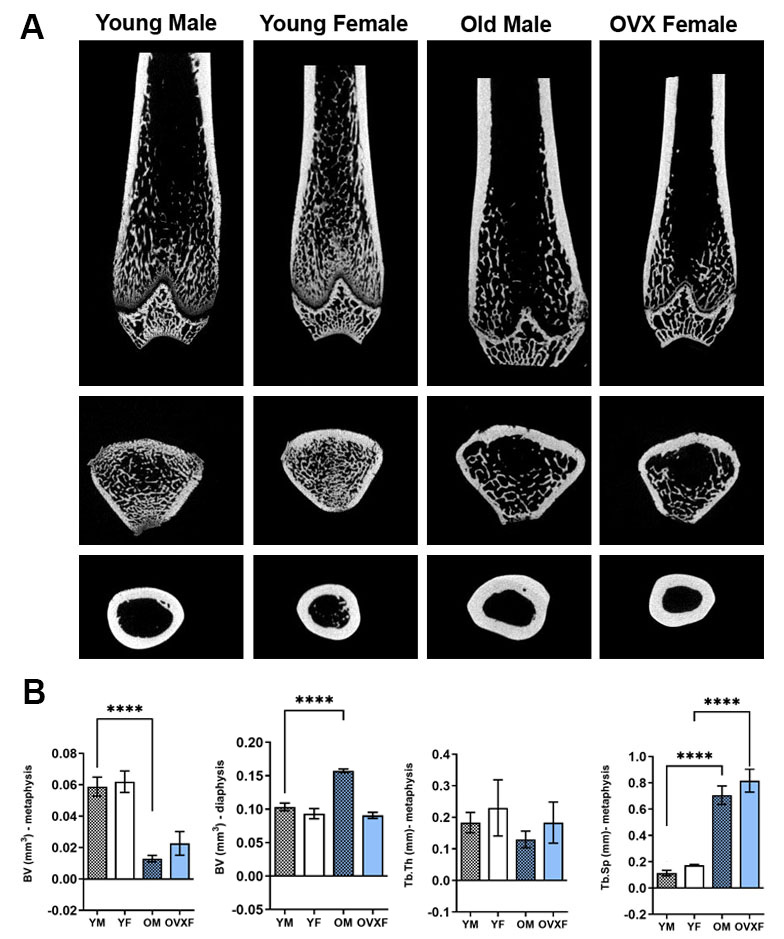

Supplement: Supplementary file 1 [file genes-13-00642-s001.zip › FigureS2_MicroCT_FinalR1.jpg]

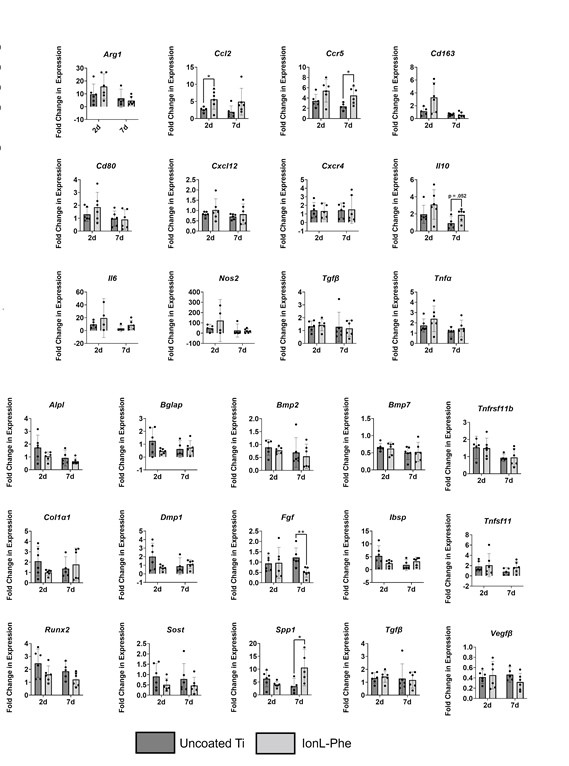

Supplement: Supplementary file 1 [file genes-13-00642-s001.zip › FigureS3_qpCRScatter_ACS_Final.tif]
